# QTL Conferring Fusarium Crown Rot Resistance in the Elite Bread Wheat Variety EGA Wylie

**DOI:** 10.1371/journal.pone.0096011

**Published:** 2014-04-28

**Authors:** Zhi Zheng, Andrzej Kilian, Guijun Yan, Chunji Liu

**Affiliations:** 1 Commenwealth Scientific and Industrial Research Organisation (CSIRO) Plant Industry, St. Lucia, Queensland, Australia; 2 School of Plant Biology, Faculty of Science and UWA Institute of Agriculture, The University of Western Australia, Perth, Australia; 3 National Foxtail Millet Improvement Centre, Institute of Millet Crops, Hebei Academy of Agricultural and Forestry Sciences, Shijiazhuang, China; 4 Diversity Arrays Technology Pty Ltd, Yarralumla, Canberra, Australia; USDA-ARS-SRRC, United States of America

## Abstract

Fusarium crown rot (FCR) is one of the most damaging cereal diseases in semi-arid regions worldwide. The genetics of FCR resistance in the bread wheat (*Triticum eastivum* L.) variety EGA Wylie, the most resistant commercial variety available, was studied by QTL mapping. Three populations of recombinant inbred lines were developed with this elite variety as the resistant parent. Four QTL conferring FCR resistance were detected and resistance alleles of all of them were derived from the resistant parent EGA Wylie. One of these loci was located on the short arm of chromosome 5D (designated as *Qcrs.cpi-5D*). This QTL explains up to 31.1% of the phenotypic variance with an LOD value of 9.6. The second locus was located on the long arm of chromosome 2D (designated as *Qcrs.cpi-2D*) and explained up to 20.2% of the phenotypic variance with an LOD value of 4.5. Significant effects of both *Qcrs.cpi-5D* and *Qcrs.cpi-2D* were detected in each of the three populations assessed. Another two QTL (designated as *Qcrs.cpi-4B.1* and *Qcrs.cpi-4B.2*, respectively) were located on the short arm of chromosome 4B. These two QTL explained up to 16.9% and 18.8% of phenotypic variance, respectively. However, significant effects of *Qcrs.cpi-4B.1* and *Qcrs.cpi-4B.2* were not detected when the effects of plant height was accounted for by covariance analysis. The elite characteristics of this commercial variety should facilitate the incorporation of the resistance loci it contains into breeding programs.

## Introduction

*Fusarium* crown rot (FCR) is a chronic and serious disease in cereal production in Australia. It has become more prevalent in many parts of the semiarid regions worldwide in recent years, due most likely to the high intensity of cereal in cropping system combined with the wide adoption of minimum tillage for moisture conservation [1]. Field surveys showed that *F*. *pseudograminearum* is the most prevalent pathogen for FCR in Queensland and New South Wales in Australia but many different species of *Fusarium* can cause this disease [2]. FCR causes an estimated annual yield loss of $79 million Australia dollars in wheat production in Australia [3]. A study in the Pacific Northwest in USA showed that FCR could reduce yields of winter wheat varieties by up to 35% [4]. Additionally, FCR infected plants may produce mycotoxins which can be harmful to humans and livestock when present in food or feed [5].

Growing resistant wheat varieties has long been recognised as a major component in minimizing FCR damage [6]. However, highly resistant wheat varieties are still not available. Over the last few years, significant efforts have been placed in identifying QTL and developing markers for breeding programs. Three commercial varieties were among the various sources of FCR resistance studied so far. Two of them are Australian varieties (Kukri and Sunco) and the other, Ernie, is a variety released in the USA. Three QTL with resistance alleles derived from these three varieties have been reported. Based on an outdoor ‘terrace’ assay, a QTL derived from Kukri was detected on chromosome 4B near the semi-dwarfing gene *Rht-B1b* [7]. Considering that QTL mapping provides only limited resolution [8] and that plant height and heading date can interfere with FCR assessments [9], [10], further studies are required to clarify the value of this QTL in breeding programs. Based on a pot assay in glasshouses, a QTL derived from Ernie was detected on chromosome arm 3BL [11]. Significant effects of this QTL were also detected in both of the validation populations used in the study [11]. A QTL with similar position and magnitude has also been detected from a highly resistant genotype belonging to *T. spelta* [12]. The same QTL may also be present in two USA varieties, Macon and Otis, although both of them were used as susceptible parents [13]. A QTL on chromosome 3B was also detected in a population of 2-49/W21MMT70 with the resistance allele derived from the male parent [14]. However, this QTL seems to be a product of specific interaction between the two parents as it was not detected in the population of W21MMT70/Mendos [15]. Analysis of a population of Sunco/2-49 using the pot assay described by Wildermuth and McNamara [16] detected a QTL with the resistance allele inherited from Sunco. This QTL was located on chromosome 2B with moderate effects [14]. Similar to many of the FCR QTL reported so far, significant effects of this QTL on 2B were not consistently detected [13].

The bread wheat variety EGA Wylie has better FCR resistance than any other varieties grown in Australia [17]. This variety was also one of the most resistant ones among the more than 2,500 genotypes screened by Liu *et al*. [18]. The combination of its elite agronomic characteristics and its level of resistance makes this variety very attractive in improving FCR resistance. To further improve the values of this variety in FCR breeding programs, we studied the genetics of its FCR resistance. Results obtained from the study are reported in this paper.

## Materials and Methods

### Plant materials

Three populations of recombinant inbred lines (RILs) for the elite variety EGA Wylie were developed in glasshouses at the Queensland Bioscience Precinct (QBP) in Brisbane, Australia. They include:

- EGA Wylie/Sumai3 RILF8: 120 lines;
- EGA Wylie/Chile RILF6: 100 lines;
- EGA Wylie/NK RILF6: 100 lines.

The three susceptible parents used in these populations have diverse origins: Sumai3 was a commercial variety released in China, Chile originated from Chile, and NK is a genotype originated from Japan. The population of EGA Wylie/Sumai3 was used for QTL mapping. The other two populations were used for validating QTL detected from the mapping population.

### Evaluation of resistance to FCR

A highly aggressive *F.pseudograminearum* isolate, CS3096, was used in FCR assessment. This isolate was collected in northern New South Wales, Australia and maintained in the CSIRO collection [2]. The methods used for inoculum preparation, inoculation and FCR assessment were based on that described by Li *et al*. [19]. Briefly, inoculum was prepared using plates of ½ strength potato dextrose agar. The inoculated plates were incubated for 7 days at room temperature before the mycelium was scraped. The plates were then incubated for a further 5–7 days under a combination of cool white and black fluorescent lights with a 12 h photoperiod. The spores were harvested and the concentration of spore suspension was adjusted to 1×10^6^ spores per millilitre in distilled water. Tween 20 was added (0.1% v/v) to the spore suspension prior to use for inoculation.

Seeds were germinated in Petri dishes on two layers of filter paper saturated with water. The germinated seedlings were immersed in the spore suspension for 1 min and two seedlings were planted into a single square punnet of a 56-well tray (Rite Grow Kwik Pots, Garden City Plastics, Australia) containing steam sterilized University of California mix C (50% sand and 50% peat v/v). The punnets were arranged in a randomized block design in a controlled environment facility (CEF). Settings for the CEF were: 25/16 (±1)°C day/night temperature and 65/85(±5)% day/night relative humidity, and a 14-hour photoperiod with 500 µmol m^−2^s^−1^ photon flux density at the level of the plant canopy. To promote FCR development, water-stress was applied during the FCR assessment. Inoculated seedlings were watered only when wilt symptoms appeared.

Three replicated trials (designated as FCR-01, FCR-02 and FCR-03, respectively) were carried out with the mapping population. Each trial contains two replicates, each with 14 seedlings. FCR severity was assessed 35 days after inoculation, using a 0 (no obvious symptom) to 5 (whole plant severely to completely necrotic) scale as described by Li *et al*. [19]. A disease index (DI) was then calculated for each line following the formula of DI = (Σ_nX_/5N)×100, where *X* is the scale value of each plant, *n* is the number of plants in the category, and *N* is the total number of plants assessed for each line.

For validating QTL detected in the mapping population, the two validation populations were assessed twice. Similar to the QTL mapping experiments, two replicates, each containing seven seedlings, were used in each of the validation trials. The 200 lines of the two validation populations were grouped into two groups based on the alleles of the most closely linked markers identified in the QTL mapping exercise. The average difference in FCR severity between the two groups was used for estimating the effect of a given QTL.

### Evaluation of plant height and heading date

To assess possible effects of plant height and heading date on FCR resistance, two field trials were conducted for the mapping population of EGA Wylie/Sumai3 at the CSIRO Research Station at Gatton in Queensland (27°34′S, 152°20′E), one in 2012 (designated as PH01 and HD01) and the other in 2013 (designated as PH02 and HD02), respectively. Randomized blocks were used for both of the field trials, each with three replicates. For each replicate, 20 seeds for each of the F8 RILs were sown in a single 1.5 m row with a 25 cm row-spacing. Five measurements were obtained from the five tallest tillers in each row and the average from the five measurements was used for statistical analyses. The heading date of a line was recorded as the day on which approximately 50% of the spikes emerged from first tillers.

### Molecular marker analysis

Leaf tissues from each of the three RIL populations were collected and stored at −80°C until processing. Genomic DNA was extracted using the CTAB method as outlined by Anderson *et al*. [20]. Genotyping based on DArT- sequencing was used for linkage map construction for the mapping population with the use of 92 RILs. DArT-sequence based genotyping of the parents and the mapping population was carried out by the Diversity Arrays Technology Pty Ltd. (http://www.triticarte.com.au). A wheat DArT-sequence array consisting of 21,362 random sequences were used. Procedures of hybridization of genomic DNA to the DArT-sequence array, image analysis and polymorphism scoring were as described by Akbari *et al*. [21]. SSR markers around each of the putative QTL were selected from the existing linkage maps [22] and used to screen the whole mapping population of 120 lines. PCR reactions for the SSR marker analyses were carried out using α [^33^P] dCTP (3,000 ci/mmol) following the manufacturer's protocol (Multiplex-Ready Marker User Handbook, version 2.0). The amplified products were mixed with an equal volume of loading dye, denatured at 95°C for 10 min, and 3.8 µl of amplified samples were separated on 5% polyacrylamide gels containing 8 M urea at 100 W for 2 hrs. The gels were subsequently dried using a gel dryer for 50 min at 80°C and exposed to Kodak X-Omat X-ray film for 4–6 days.

### Data analysis and QTL mapping

Statistical analyses were performed using GenStat for Windows, 13th edition (copyright Lawes Agricultural Trust, Rothamsted Experimental Station, UK) and the SPSS statistics 17.0 for Windows statistical software package (SPSS Inc., Chicago, IL). For each trial, the following model of mixed-effects was used: Yij = *µ*+*ri*+*gj*+*wij*, where: Yij = trait value on the *j*th genotype in the *i*th replication; *µ* = general mean; *ri* = effect due to *i*th replication; *gj* = effect due to the *j*th genotype; *wij* = error or genotype by replication interaction, where genotype was treated as a fixed effect and that of replicate as random. The effects of replicate and genotype for each trait were determined using ANOVA. The Pearson correlation coefficient was estimated between the traits and trials.

Linkage analysis was carried out using the computer package JoinMap 4.0 [23]. LOD thresholds from 3 to 10 were tested, until a threshold with the optimum number of markers in linkage groups maintaining linkage order and distance was obtained. MapQTL 5.0 [24] was used for QTL analysis. The Kruskal–Wallis test was used in a preliminary testing of associations between markers and FCR severity. Interval mapping (IM) was used to identify QTL. Automatic cofactor selection was used to fit the multiple QTL model (MQM) (backward elimination at *P*>0.02) and to select significantly associated markers as cofactors. For each trial, a test of 1,000 permutations was performed to identify the LOD threshold corresponding to a genome-wide false discovery rate of 5% (*P*<0.05). Based on the permutation test, a threshold LOD value was used to declare the presence of a QTL. A linkage map showing the QTL positions was drawn using MapChart [25].

## Results

### Segregation of FCR resistance in the mapping population of EGA Wylie/Sumai3

The average FCR ratings for the three trials conducted in this study were similar (53.96 for FCR-01, 51.67 for FCR-02, and 53.86 for FCR-03), although the range of ratings for individual lines was wider in FCR-03 (Table 1). Correlation coefficients (*R^2^*) among the three trials were significant and positive with *R^2^* ranging from 0.59 to 0.95 (Table 2).

**Table 1:** Disease index (DI) of Fusarium crown rot (FCR) and plant height in the population EGA Wylie/Sumai3#_._

Traits		Trial	Population			
			Min	Max	Mean	SD
FCR (DI)		FCR-01	37.55	74.50	53.96	8.68
		FCR-02	34.97	74.50	51.67	8.09
		FCR-03	34.10	80.27	53.86	9.77
Plant height (cm)		PH-01	50.80	110.00	81.28	14.35
		PH-02	46.80	116.00	84.04	15.09

# The three trials conducted for assessing FCR severity were designated as FCR-01, FCR-02, and FCR-03, respectively; and the two trials conducted for estimating plant height (PH) were designated as PH-01 and PH-02, respectively.

**Table 2:** Correlation coefficients of FCR resistance in the population of EGA Wylie/Sumai3 among the three replicated trials#_._

Trial	FCR-01	FCR-02	FCR-03
FCR-01	1.00		
FCR-02	0.59**	1.00	
FCR-03	0.95**	0.60**	1.00

# The three trials conducted for assessing FCR severity were designated as FCR-01, FCR-02 and FCR-03, respectively. ‘**’ indicates significant at *p*<0.01.

### Linkage map constructed and QTL for FCR resistance detected in the mapping population

Of the DArT-sequences analysed, 1,720 high quality polymorphic markers between the two parents of the mapping population, EGA Wylie and Sumai3, were selected for linkage map construction (Table S1). Based on positions of putative QTL identified for FCR resistance, 24 SSR markers were then inserted into the linkage map. These DArT-sequences and SSR markers formed 61 linkage groups and covered a total genetic distance of 2,063 cM with an average distance of 1.2 cM between loci.

**Figure 1:**
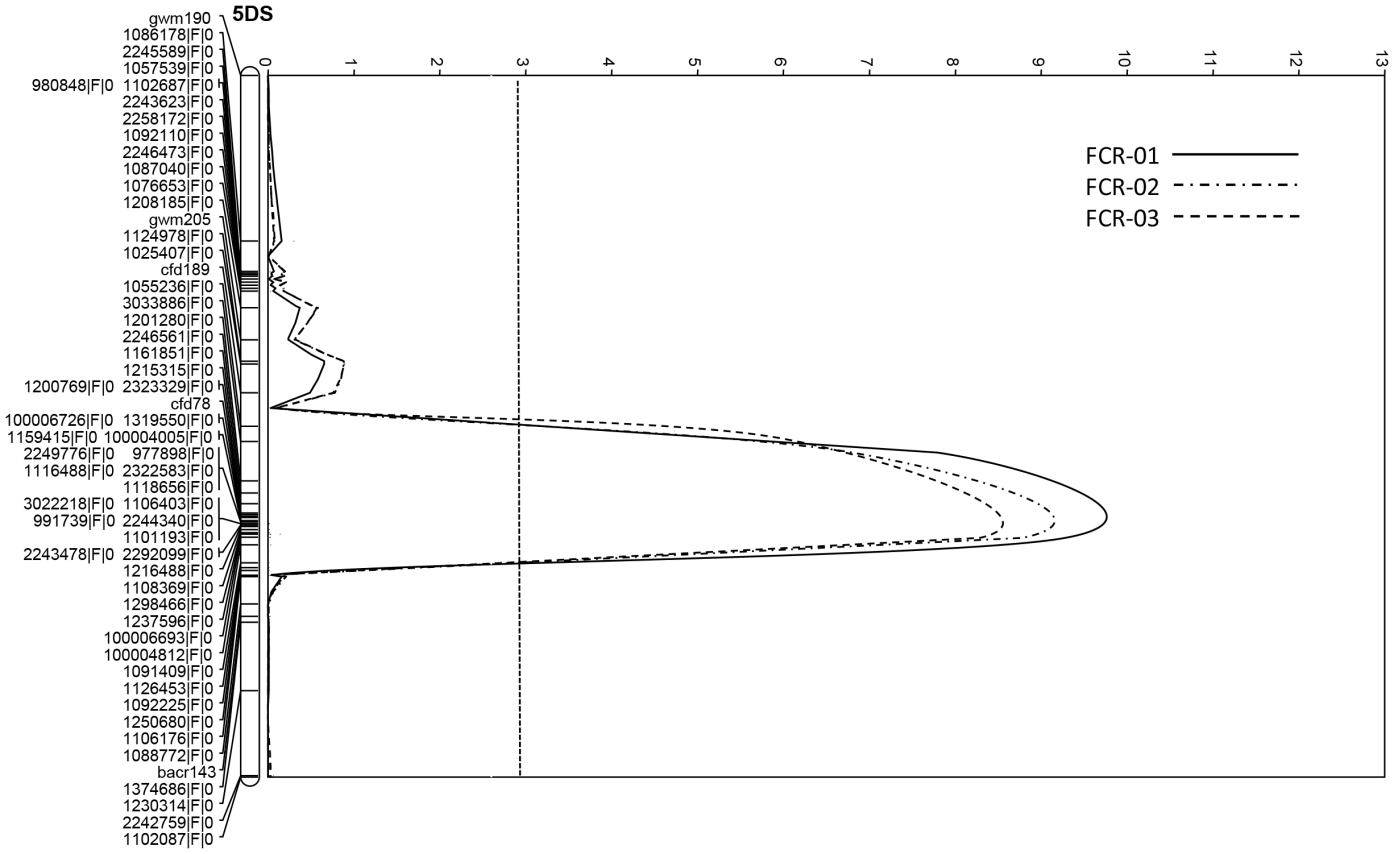
QTL conferring FCR resistance detected with interval mapping (IM) on chromosome 5D in the population of EGA Wylie/Sumai3. The LOD values from each centimorgan of the chromosome were plotted against the chromosome, and the threshold LOD value (2.9) based on permutation test for declaring the presence of a QTL was indicated by a dotted line.

**Figure 2:**
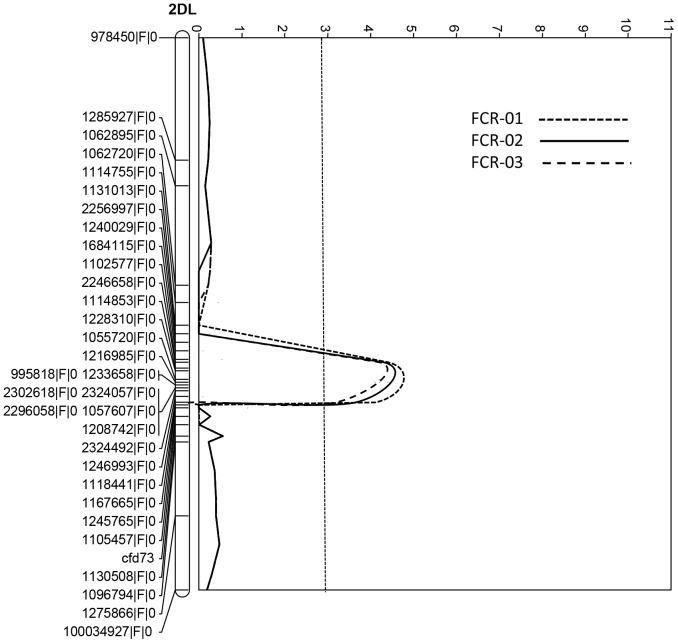
QTL conferring FCR resistance detected with interval mapping (IM) on chromosome arm 2DL in the population of EGA Wylie/Sumai3. The LOD values from each centimorgan of the chromosome were plotted against the chromosome, and the threshold LOD value (2.9) based on permutation test for declaring the presence of a QTL was indicated by a dotted line.

**Figure 3:**
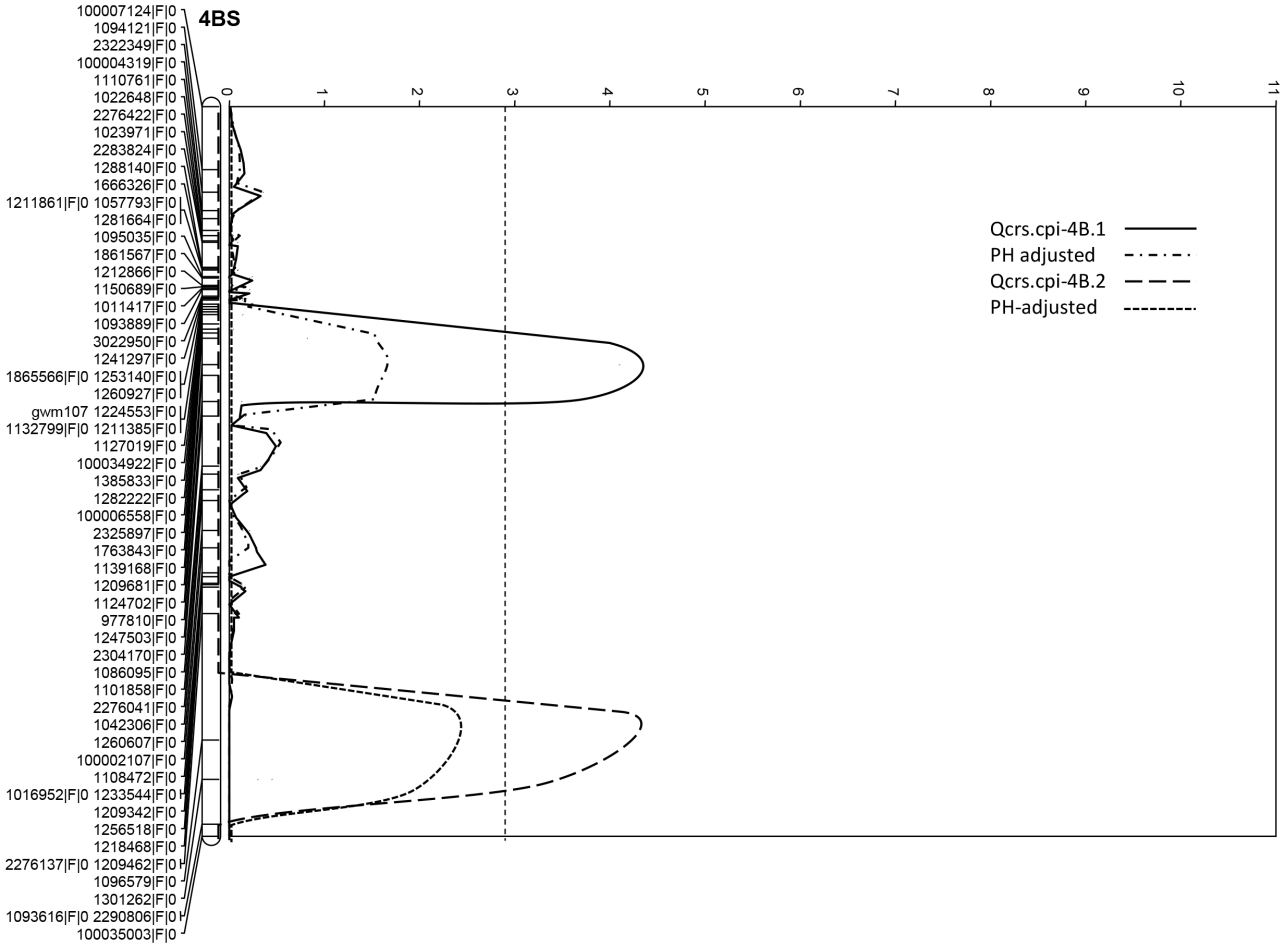
LOD values of *Qcrs.cpi-4B.1* and *Qcrs.cpi-4B.2* obtained from combined data of the three FCR trials pre- (combined) and post-adjustment by plant height (PH-adjusted).

Permutation tests found that a LOD score of 2.9 was the threshold for all the three trials. Based on this threshold, four QTL conferring FCR resistance were identified in each of the three trials. All of them were derived from the resistant parent EGA Wylie. The most significant QTL, designated as *Qcrs.cpi-5D*, was identified on the short arm of chromosome 5D. It explained up to 31.1% of the phenotypic variance with a LOD score of 9.6 (Table 3; Figure 1). The second QTL was mapped on the long arm of chromosome 2D. This QTL, designated as *Qcrs.cpi-2D*, was also detected in all three trials and explained up to 20.2% of phenotypic variance with a LOD value of 4.5 (Table 3; Figure 2). Two more QTL, designated as *Qcrs.cpi-4B.1* and *Qcrs.cpi-4B.2*, respectively, were located on the short arm of chromosome 4B. These two QTL explained up to 16.5% and 18.8% of the phenotypic variance with the LOD value of 4.3 and 4.2, respectively (Table 3). The distance between these two QTL on chromosome 4B is approximately 28 cM (Figure 3).

**Table 3:** QTL for FCR severity, plant height and heading date identified in the population of EGA Wylie/Sumai3#_._

Trials	Analysis	Chromosome	Flanking markers	LOD	R^2^(%)
FCR-01	IM	5DS	1215315|F|0 & 1237596|F|0	9.6	31.1
		4BS.1	100004319|F|0 & 2324159|F|0	4.3	16.9
		4BS.2	1108472|F|0 & 1093616|F|0	4.2	18.7
		2DL	1131013|F|0 & 1246993|F|0	4.2	18.8
	MQM	5DS	1201280|F|0 & 100004812|F|0	9.2	30.0
		4BS.1	2322349|F|0 & 1023971|F|0	4.2	15.5
		4BS.2	1108472|F|0 & 2290806|F|0	4.2	16.5
		2DL	1114755|F|0 & 1228310|F|0	3.6	16.3
FCR-02	IM	5DS	1215315|F|0 & 1237596|F|0	9.0	29.4
		4BS.1	100004319|F|0 & 2324159|F|0	3.8	14.8
		4BS.2	1108472|F|0 & 1093616|F|0	3.9	17.2
		2DL	1131013|F|0 & 1246993|F|0	4.4	19.8
	MQM	5DS	1201280|F|0 & 100004812|F|0	8.6	28.2
		4BS.1	2322349|F|0 & 1023971|F|0	3.6	13.9
		4BS.2	1108472|F|0 & 2290806|F|0	3.7	15.6
		2DL	1114755|F|0 & 1228310|F|0	3.7	16.9
FCR-03	IM	5DS	1215315|F|0 & 1237596|F|0	8.6	28.4
		4BS.1	100004319|F|0 & 2324159|F|0	3.8	15.1
		4BS.2	1108472|F|0 & 1093616|F|0	3.9	17.1
		2DL	1131013|F|0 & 1246993|F|0	4.5	20.2
	MQM	5DS	1201280|F|0 & 100004812|F|0	8.2	27.1
		4BS.1	2322349|F|0 & 1023971|F|0	3.5	14.2
		4BS.2	1108472|F|0 & 2290806|F|0	3.8	15.6
		2DL	1114755|F|0 & 1228310|F|0	3.8	17.1
PH-01	IM	4BS	2283824|F|0 & 1211861|F|0	18.9	64.3
	MQM	4BS	2283824|F|0 & 1763843|F|0	17.5	58.7
PH-02	IM	4BS	2283824|F|0 & 1211861|F|0	14.7	54.9
	MQM	4BS	2283824|F|0 & 3027835|F|0	13.3	48.9
HD-01	IM	2DS	1302829|F|0 & 2249013|F|0	7.3	35.7
	MQM	2DS	1302829|F|0 & 2249013|F|0	7.3	33.0
HD-02	IM	2DS	1302829|F|0 & 2249013|F|0	7.6	36.5
	MQM	2DS	2249013|F|0 & 1110431|F|0	7.4	34.5

# The three trials conducted for assessing FCR severity were designated as FCR-01, FCR-02 and FCR-03, respectively; the two trials conducted for estimating plant height were designated as PH-01 and PH-02, respectively; and the two trials conducted for estimating heading date were designated as HD-01 and HD-02, respectively. IM = interval mapping; and MQM = QTL detection with a multiple QTL model.

### Effects of plant height and heading date on FCR resistance

A QTL controlling heading date was identified on the short arm of chromosome 2D.

This QTL explained up to 36.5% of phenotypic variance with a LOD value of 7.6 (Table 3). A QTL affecting plant height was detected on the short arm of chromosome 4B. This QTL explained up to 64.3% of the phenotypic variance with a LOD value of 18.9 (Table 3). Interactions between FCR resistance and heading date were not significant based on the co-factor analysis conducted. However, significant interactions between FCR resistance and plant height were detected (Table 4). Plant height had little effects on *Qcrs.cpi-5D* and *Qcrs.cpi-2D* but neither *Qcrs.cpi-4B.1* nor *Qcrs.cpi-4B.2* remained significant when the effect of plant height was taken into account by the co-factor analysis (Figure 3). Thus, these two QTL were not further assessed in the two validation populations.

**Table 4:** Correlation coefficients between FCR severity, plant height and heading date among five trials of the EGA Wylie/Sumai3 population#_._

Trial	FCR-01	FCR-02	FCR-03
PH-01	0.20^*^	0.18^*^	0.18^*^
PH-01	0.22^*^	0.19^*^	0.19^*^
HD-01	−0.08	−0.09	−0.08
HD-02	−0.07	−0.07	−0.06

# The three trials conducted for assessing FCR severity were designated as FCR-01, FCR-02 and FCR-03, respectively; the two trials conducted for estimating plant height were designated as PH-01 and PH-02, respectively; and the two trials conducted for estimating heading date were designated as HD-01 and HD-02, respectively.‘*’ indicates significant at *p*<0.05.

### Effects of FCR resistant QTL in different genetic backgrounds

For assessing the possible effects of *Qcrs.cpi-5D* and *Qcrs.cpi-2D*, SSR markers linked closely to these two loci (cfd78 and cfd73, respectively) were used to genotype the two validation populations. Based on the presence or absence of alleles from the resistant parent EGA Wylie, the 200 RILs were placed into two groups. Significant differences in DI between the two groups of RILs were detected for both of the QTL in each of the two populations. The average difference for *Qcrs.cpi-5D* was 29.6% in the population of EGA Wylie/Chile and 22.2% in the population of EGA Wylie/NK. The average difference between DI values of the two groups of lines for *Qcrs.cpi-2D* was 16.2% in the population of EGA Wylie/Chile and 13.1% in the population of EGA Wylie/NK (Table 5).

**Table 5:** Effects of QTL conferring FCR resistance in the two validation populations#_._

Population	QTL	RR	rr	Difference (%)	*p* value
EGA Wylie/Chile	*Qcrs.cpi-5D*	23.2	33.1	29.6	*p*<0.01
	*Qcrs.cpi-2D*	24.8	29.6	16.2	*p*<0.01
EGA Wylie/NK	*Qcrs.cpi-5D*	30.1	38.7	22.2	*p*<0.01
	*Qcrs.cpi-2D*	24.6	28.3	13.1	*p*<0.01

# ‘RR’ represents homozygous alleles from the resistant parent EGA Wylie, and ‘rr’ those from non-' EGA Wylie' parents.

## Discussion

EGA Wylie is an elite variety with the best FCR resistance among all available varieties in Australia [17]. It was also one of the most resistant genotypes found in a systematic screening of over 2,500 lines [18]. For investigating the genetics of its FCR resistance, three RIL populations were developed for this variety using susceptible parents with diverse origins. QTL mapping determined that the FCR resistance of EGA Wylie was conditioned by four loci. Of these, the one on 5DS explained up to 31.1% of phenotypic variance and the one on 2DL explained up to 20.2% of phenotypic variance. Significant effects of both of these loci were also detected in each of the two validation populations assessed in this study. Significant effects for the other two loci, both located on chromosome arm 4BS, were not detected when the effects of plant height were taken into consideration by a co-factor analysis. Thus further studies are needed to clarify the values of the two QTL on 4BS in FCR resistance breeding. The two QTL on 5DS and 2DL, however, can be highly valuable for FCR resistance breeding. Together with the one on 3BL [11]–[14], they are the only three FCR loci reported so far which have been consistently detected in different genetic backgrounds. The elite characteristics of the commercial variety EGA Wylie mean that incorporating these resistant loci into breeding programs should not be difficult, while incorporating other loci of resistance into this elite variety may also be a practical approach in FCR breeding.

Analysing a double haploid population between 2-49 and W21MMT70 detected two FCR loci on chromosomes 5D and 2D, respectively. The resistance alleles of both loci were derived from the genotype W21MMT70 [14]. However, neither of the QTL was consistently detected in the three assays conducted in that study and their effects in other genetic backgrounds have never been assessed. Further, these two QTL seem to be the products of the specific interaction between the two parents of the mapping population as neither of them were detected in a population between W21MMT20 and Mendos [15]. As QTL mapping provides only limited resolution [8], the available data are not adequate in determining whether the FCR resistant loci on 5D and 2D detected in 2-49/W21MMT70 are the same as those detected in this study. It is of interest to note that W21MMT70 originated from Western Australia where FCR is not yet a major threat to wheat production [3].

The significant interaction between plant height and the two FCR loci on 4BS is another example demonstrating the necessity of examining traits of agronomic importance when studying disease resistance. Significant interactions between plant height and FCR resistance have been reported in both wheat [9] and barley [26], [27], with difference in cell density between the tall and short genotypes being hypothesized as a likely contributing factor. Similar interactions between plant height and Fusarium head blight have also been reported in wheat, with the difference in microclimate exposed by spikes of tall and short plants as a likely contributing factor to Type I resistance [28]. Thus, before trying to incorporate them into breeding programs, further studies are required to determine the values of the two FCR loci on 4BS.

The susceptible parent Sumai3 used in the mapping population in this study has been widely used as a major source of resistance for Fusarium head blight (FHB) worldwide [29], [30]. Analysing this genotype or its derivatives has identified several QTL conferring FHB resistance. Three of these QTL, located on 3BS, 5A and 6B, respectively, have been repeatedly detected in a wide range of genetic backgrounds and environments [29]. However, none of these loci was detected for FCR resistance in this study. The lack of genetic association between genes conferring resistance to these two diseases was also reported in the study of Ernie, a winter wheat from the USA with resistance to both diseases. QTL conferring resistance to these two diseases in Ernie were also located on different chromosomes [11]. These results reinforce the viewpoint that, although FCR and FHB can be caused by the same pathogens and share the common aetiology [1], the large quantity of data accumulated from FHB research may have only limited value for FCR research and separate screening may have to be conducted in identifying genotypes with high quality of FCR resistance.

## Supporting Information

Table S1 **DArT-sequences for generating the 1,720 polymorphic markers used in this study.**
(XLSX)
